# A Novel Approach for Prediction of Vitamin D Status Using Support Vector Regression

**DOI:** 10.1371/journal.pone.0079970

**Published:** 2013-11-26

**Authors:** Shuyu Guo, Robyn M. Lucas, Anne-Louise Ponsonby, the Ausimmune Investigator Group

**Affiliations:** 1 National Centre for Epidemiology and Population Health, The Australian National University, Canberra, Australia; 2 Murdoch Childrens Research Institute, Melbourne, Australia; UMR-S665, INSERM, Université Paris Diderot, INTS, France

## Abstract

**Background:**

Epidemiological evidence suggests that vitamin D deficiency is linked to various chronic diseases. However direct measurement of serum 25-hydroxyvitamin D (25(OH)D) concentration, the accepted biomarker of vitamin D status, may not be feasible in large epidemiological studies. An alternative approach is to estimate vitamin D status using a predictive model based on parameters derived from questionnaire data. In previous studies, models developed using Multiple Linear Regression (MLR) have explained a limited proportion of the variance and predicted values have correlated only modestly with measured values. Here, a new modelling approach, nonlinear radial basis function support vector regression (RBF SVR), was used in prediction of serum 25(OH)D concentration. Predicted scores were compared with those from a MLR model.

**Methods:**

Determinants of serum 25(OH)D in Caucasian adults (n = 494) that had been previously identified were modelled using MLR and RBF SVR to develop a 25(OH)D prediction score and then validated in an independent dataset. The correlation between actual and predicted serum 25(OH)D concentrations was analysed with a Pearson correlation coefficient.

**Results:**

Better correlation was observed between predicted scores and measured 25(OH)D concentrations using the RBF SVR model in comparison with MLR (Pearson correlation coefficient: 0.74 for RBF SVR; 0.51 for MLR). The RBF SVR model was more accurately able to identify individuals with lower 25(OH)D levels (<75 nmol/L).

**Conclusion:**

Using identical determinants, the RBF SVR model provided improved prediction of serum 25(OH)D concentrations and vitamin D deficiency compared with a MLR model, in this dataset.

## Introduction

There have been increasing concerns about vitamin D deficiency around the world. Epidemiological evidence suggests that hypovitaminosis D is linked to various chronic diseases such as colorectal, prostate and breast cancers[1], [2], [3], as well as cardiovascular diseases and diabetes[4], [5], [6]. Vitamin D status is assessed by the serum concentration of 25-hydroxyvitamin D (25(OH)D), an accepted biomarker[7]. However measuring 25(OH)D requires blood sampling and laboratory resources for quantitative assays. This approach may not be feasible for testing hypotheses of vitamin D status as a risk factor for chronic disease in large epidemiological studies.

An alternative approach for estimating vitamin D status is to derive a predictive model based on measurements of 25(OH)D concentration and questionnaire data on known determinants, from a subset of the study cohort. Values for the remainder of the cohort are then predicted, based on their questionnaire data[8], [9], [10]. Past studies have used multiple linear regression (MLR) modelling to develop these predictive models. However, the final models typically explain only a small proportion of the total variability in 25(OH)D concentration, that is, the coefficient of determination (R^2^) values from such predictive models have ranged from 0.13 to 0.42[8], [9], [10], [11], [12], [13]. In some publications, predicted and actual 25(OH)D levels have been compared in a validation sample, with Spearman(9,10) or Pearson(12) correlation coefficients ranging from 0.23 to 0.51.

Recent studies on vitamin D status prediction are shown in **Table 1**. These models, based on MLR, have a number of potential limitations. For example, outliers can be highly influential in MLR models, with large differences in parameters dependent on inclusion or exclusion of these values. Moreover, MLR reflects a relationship between the means of the dependent variable and the independent variables[14], although in chronic disease epidemiology, we may be most interested in very low 25(OH)D values. Thus the 25(OH)D scores predicted using MLR models may not accurately reflect an individual's actual vitamin D status, biasing any risk factor associations. Nevertheless, vitamin D prediction models could have considerable potential, both in studies examining vitamin D status in relation to disease risks and in screening for risk of vitamin D deficiency and thus the need for testing – but require improved prediction accuracy. Newer modelling techniques may provide better fit and more accurate assignment of participants to categories of vitamin D status, e.g. deficient, insufficient, sufficient, or optimal.

**Table 1:** Recent studies using a multiple linear regression prediction model for 25(OH)D concentration.

Reference	Cohort	Sample	Model covariates	R^2^ for the model	Validation
Giovannucci et al,	Health Professionals	Male	Geographical region	28%	Measured plasma 25(OH)D level rose across increasing
2006 [8]	Follow-Up Study (HPFS),	40–75	Dietary vitamin D intake		deciles of predicted 25(OH)D score (p_trend_<0.001)
	US	Training set: 1095	Vitamin D supplements		
		Validation set: 542	Race		
			BMI		
			Physical activity level		
Chan et al., 2010	Adventist Health Study-2	Male & Female	Race	White: 22%	N/A
[11]	(AHS-2),	Black: 209	BMI	Black: 31%	
	US, Canada	White: 236	Skin type	Total: 42%	
			UV season		
			Latitude		
			Erythemal zone		
			Total vitamin D intake		
			Duration of sun exposure		
			Percentage of body exposed		
Liu et al., 2010	Framingham Offspring	Male & Female	Age	25.75%	Spearman rho for measured 25(OH)D concentration vs.
[9]	Study,	50–70	Sex		predicted score = 0.51 (p<0.001)
	Massachusetts, US	Training set: 883	BMI		
		Validation set: 845	Total vitamin D intake		
			Smoking status		
			Total energy intake		
Millen et al.,	Women's Health Initiative	Female	Langleys	21%	Pearson correlation coefficient for measured plasma
2010	Clinical Trial (WHI-CT),	50–79	Race		25(OH)D vs. predicted score r = 0.45, 95%CI: 0.40,0.49
[12]	US	Training set: 3055	Age		The predictive model was poor at categorizing women in the
		Validation set: 1528	Waist circumference		severely deficient (3%) and sufficient (3%) range of vitamin
			Recreational physical activity		D status.
			Total vitamin D intake		
Peiris et al., 2011	Veterans Administration	Male	Triglyceride	12.9%	The model correctly classified vitamin D deficiency status
[13]	Center patients		Race		for 70.6% patients; only 30.6% of those who were actually
	Southeastern US		Total cholesterol		deficient were correctly identified as deficient.
			BMI		
			Calcium level		
			Number of missed appointments		
Bertrand et al.,	Nurses' Health Study	NHS: female, 30–55 y	Race	NHS: 33%	Spearman rho for measured 25(OH)D concentration vs.
2012 [10]	(NHS)	Training set:2246	UV-B flux	NHSII: 25%	predicted score were 0.23, 95%CI: 0.16,0.29 for NHS, 0.42,
	Nurses' Health Study II	Validation set:818	Dietary vitamin D intake	HPFS: 28%	95%CI:0.34, 0.49 for NHSII, 0.30, 95%CI: 0.21 0.37
	(NHSII)	NHSII: female, 25–42 y	Supplementary vitamin D intake		(adjusted for batch, age and season of blood draw)
	Health Professionals	Training set:1646	BMI		
	Follow-up Study (HPFS)	Validation set: 479	Physical activity		
		HPFS: Male, 40–75 y	Alcohol intake		
		Training set: 1255	Post-menopausal hormone use		
		Validation set: 841	Season of blood draw		

### Support vector regression (SVR) algorithm

Data modelling methods based on machine learning, such as Artificial Neural Networks (ANN) and Support Vector Machines (SVM), have been extensively used in bioinformatics and molecular biology[15], . More recently, these techniques have been introduced to solve medical classification and medical prediction problems and aid clinical decision making[18], [19], [20], [21]. In the epidemiology domain, machine learning algorithms also have the potential for prediction, classification and risk factor identification. For example, this type of modeling has been used for risk prediction of common diseases such as diabetes and pre-diabetes[22].

The SVM algorithm was originally developed by Vapnik and co-workers at AT&T Bell Laboratories in the 1990s[23], [24]. The underlying theory and algorithm were introduced by Elisseeff *et al.* [25]. SVM methods include support vector classification (SVC) for classification and support vector regression (SVR) for prediction.

**Figure 1:**
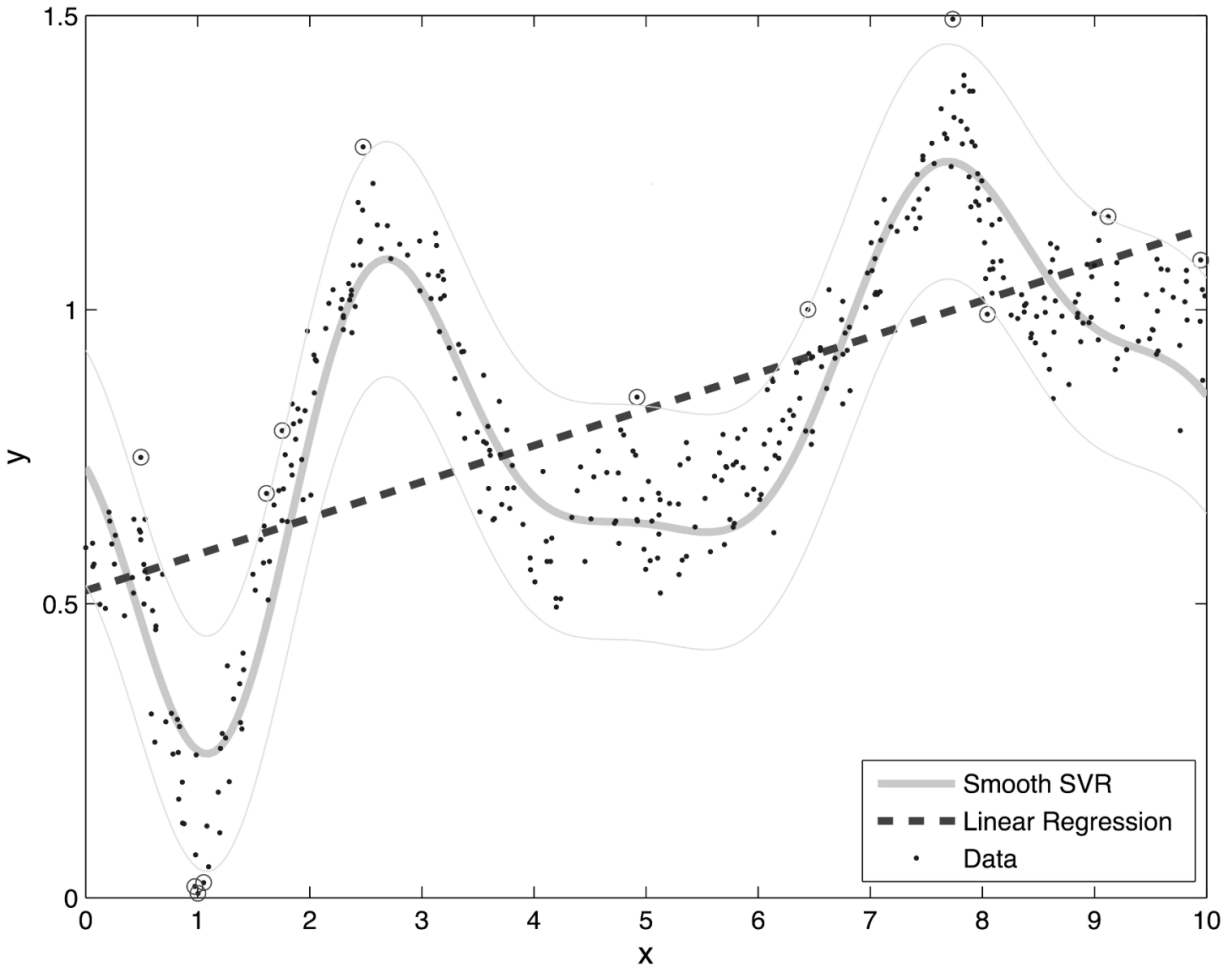
Performance demonstration of SVR and MLR in a simple scenario (two-dimensional case). The black dots indicate actual simulation data set. The solid curve denotes SVR regress line and the dot line represents the MLR regression line. The simulation data set is randomly generated by MATLAB.

The SVR method differs from that of MLR in the underlying theoretical settings. The basic idea of regression methods is to construct an optimal regression hyperplane with n-1 dimensions that best fits the data in an n-dimensional space. If we take the simplest example, a two-dimensional data space can be generated by two variables in a dataset; the regression hyperplane is a straight line (with one dimension). As for other conventional methods, the MLR algorithm fits a model using the least mean squares approach to define the linear hyperplane[26], [27]. However, the real world is much more complicated than a linear correlation. Furthermore, the regression hyperplane based on a least mean squares approach is greatly affected by outliers. In the SVR method, these problems are solved by 1) using integrating kernel functions (i.e polynomial, sigmoid and radial basis functions) to add more dimensions to lower dimensional space or add nonlinearity to the model; and 2) introducing user-specified parameters to control the trade-off of prediction errors and flatness of the regression plane (see Methods section). **Figure 1** illustrates the difference between MLR and SVR prediction models.

In this paper, we examine the utility of an SVR algorithm, in comparison with a MLR algorithm, in predicting serum 25(OH)D concentration based on the determinants of vitamin D status already identified in a population of Australian Caucasian adults.

## Materials and Methods

### Study population

Data included here are from 494 participants from the control group of the Ausimmune Study[28]. The Ausimmune Study is a multi-centre, case-control study examining risk factors for multiple sclerosis. The control group was randomly selected from the Australian Electoral Roll in four different study regions. Participants completed a questionnaire including self-reported recent sun exposure and sun protection behaviours, physical activity, smoking history, diet and the use of supplements. Skin types were defined by spectrophotometric measurements of skin reflectance to calculate melanin density for exposed skin sites (dorsum of hand, shoulder) and non-exposed skin sites (upper inner arm, buttock) using a spectrophotometer (Minolta 2500d)[29]. Height, weight, waist and hip circumference were also measured. Serum 25(OH)D levels were determined by liquid chromatography dual mass spectrometry at a central laboratory. Because the number of non-Caucasian participants was small (n = 26), only data from the Caucasian participants in the control group were included for the purpose of developing the vitamin D prediction model.

### Statistical analysis

#### The MLR model

The important determinants of vitamin D status were defined using MLR and forward purposeful selection of covariates, as previously described[30]. Briefly, 12 variables were retained in the MLR environmental and phenotypic determinants model: latitude, ambient ultraviolet radiation levels, ambient temperature, hours in the sun 6 weeks before the blood draw (log transformed to improve the linear fit), frequency of wearing shorts in the last summer, physical activity (three levels: mild, moderate, vigorous), sex, hip circumference, height, left back shoulder melanin density, buttock melanin density and inner upper arm melanin density. A square root transformation of the dependent variable (serum 25(OH)D concentration) in the MLR model was performed because of heteroscedasticity of the residuals[30].

#### The SVR model

Given a dataset with *n* independent variables and *m* observations, the MLR model can be written as y = ƒ(*x*) = *W.X* +b where *W* represents the vector of the coefficients, *X* represents the vector of the independent variables, and b is the intercept. To estimate the best fit, we minimize the sum of the squared errors:



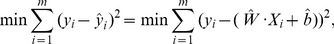
(where *i* represents the *i* ^th^ observation).

When the correlation between *x* and *y* is linear, the form of the SVR algorithm is similar to that of MLR: y = ƒ(*x*) = *W.X* +b. However, the SVR method has two additional parameters: *C* and ε. The parameter *C* is introduced to adjust the error sensitivity of the training data in order to avoid over-fitting; setting *C* to a high value results in fewer prediction errors in the training data:



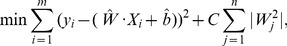
(where *j* represents the *j* ^th^ variable), The second parameter ε is the regularization constant, which controls the flatness of the final model [31].The goal of SVR is to determine an optimal function that has less than ε deviation from the target values for the training data, so that we do not count errors that are less than ε, and at the same time the regression hyperplane needs to be as flat as possible.

By using different kernel functions, which transform data into a high dimensional space or add non-linearity, the SVR algorithm allows application of nonlinear regression[32]. The Radial Basis Function (RBF) SVR method adopts the RBF kernel function, also known as the Gaussian kernel, which is the same as a Gaussian distribution function. Compared to linear SVR, the RBF SVR method has one more parameter, γ, which determines the degree of nonlinearity[33].

For the RBF SVR modelling, the data were randomly separated into two independent samples: the ‘training sample’ (n = 294) was used to develop the parameters of the vitamin D prediction model and the ‘validation sample’ (n = 174) was used for all statistical analyses noted below. The same 12 variables were included in the model as for the MLR modelling, described above. Parameters were determined by grid search, i.e. exhaustive searching through a set of parameters, followed by cross validation. The parameters with the best model performance were selected.

#### Model comparison

Predicted values from the MLR model were derived by summing coefficients multiplied by the individual values of the covariates[8]. Predicted values from the SVR model were derived by running the model with the individual values of the covariates. We compared the predictions from the RBF SVR and MLR models to measured 25(OH)D values in the “validation sample” Results were reported as means, standard deviations (SDs), minima and maxima. Mean absolute differences, i.e. the mean of the absolute differences between the individual predicted and measured 25(OH)D values, were calculated as an indication of the magnitude of error. Differences between results from the RBF SVR and MLR models were analysed with the Wilcoxon signed rank test. The correlation between predicted and measured serum 25(OH)D concentrations was analysed using a Pearson correlation coefficient (r). Bland-Altman plots were used to provide the mean bias (the average of the difference between measured 25(OH)D and prediction scores from the two compared modelling methods) across the range of 25(OH)D levels, and 95% limits of agreement between the methods.

We tested the accuracy of classification into categories of vitamin D status using predicted 25(OH)D scores. Data in the validation sample were analysed by generating the receiver operating characteristic (ROC) curve. Sensitivities and specificities were generated for a range of cut offs for the ROC curve. In chronic disease epidemiology studies, “exposures” are often categorised into quintiles. Thus, here individuals in the validation set were also classified according to quintile of predicted 25(OH)D scores and measured 25(OH)D concentration, for the purpose of testing the performance of the two models.

Data analysis for the RBF SVR model was performed using Matlab R2001b. Analyses for the MLR model, Pearson correlation, Wilcoxon signed rank test, Bland-Altman plots and ROC curves were performed using Stata 12.0 (Statacorp, Texas).

## Results

**Figure 2:**
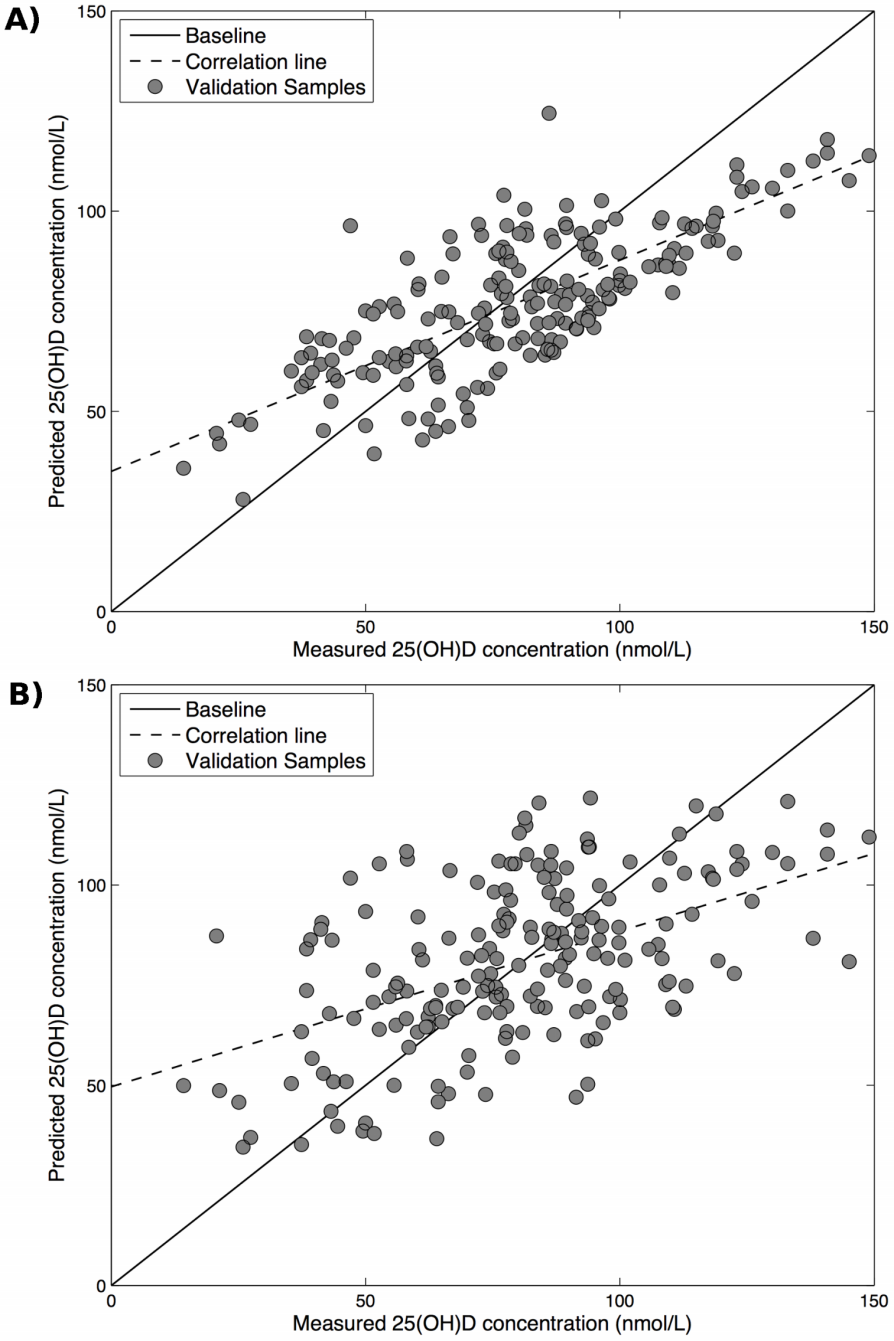
Correlation of measured 25(OH)D concentration (nmol/L) and predicted 25(OH)D concentration using (A) a multiple linear regression model; and (B) a radial basis function support vector regression model.

**Figure 3:**
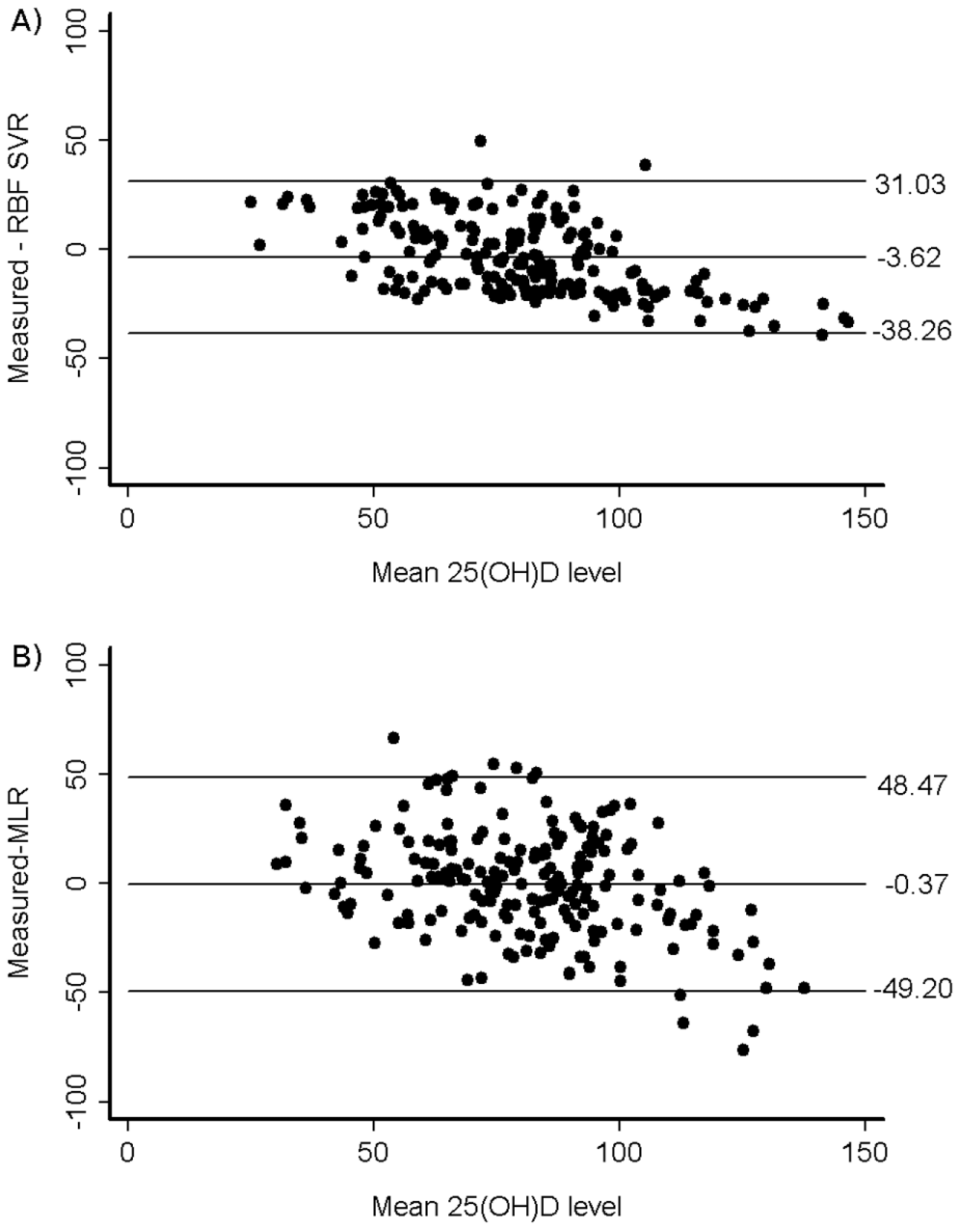
Bland – Altman plots of measured 25(OH)D concentration compared to predicted scores from (A) a MLR model; (B) a RBF SVR model. The solid lines indicate the mean bias (middle line) and 95% limits of agreement (top and bottom lines). All measurements are in nmol/L.

Means, SDs, minima and maxima of predicted 25(OH)D scores for the two models are presented in **Table 2**. A summary, as the mean absolute difference between measured and predicted 25(OH)D for the two models, is also given. The mean absolute difference between measured and predicted 25(OH)D concentrations generated by the RBF SVR model was significantly smaller than that for the MLR model (p = 0.012). Figure 2 demonstrates the correlation between the measured and predicted 25(OH)D concentration for the MLR (Figure 2A) and RBF SVR (Figure 2B) models. Consistent with this, the Pearson correlation coefficients indicated better correlation between predicted scores and measured 25(OH)D concentrations for the RBF SVR model (r = 0.74) than for the MLR model (r = 0.51). Bland Altman plots showed that there was tighter agreement between measured 25(OH)D concentration and predicted scores for the RBF SVR model than for the MLR model: 95% limits of agreement were −49.20, 48.37 (Figure 3A) and −38.26, 31.03 (Figure 3B) for the MLR and RBF SVR models, respectively. There was a slight negative bias across the range of measured 25(OH)D concentrations that was greater for the RBF SVR than the MLR predicted scores (−3.62 nmol/L, −0.37 nmol/L, respectively). Predicted scores from both models showed a greater tendency to negative bias at higher 25(OH)D concentrations.

**Table 2:** Predicted 25(OH)D concentration and mean absolute difference between predicted and measured 25(OH)D level (nmol/L).

	Mean	Standard deviation	Minimum	Maximum
Measured 25(OH)D level	81.71	28.33	14.2	163.3
Predicted level MLR	81.3	20.41	34.54	121.71
Predicted level RBF SVR	78.10	18.87	28.01	129.91
Mean absolute difference MLR	19.04	15.23	0.18	76.39
Mean absolute difference RBF-SVR	15.65	8.91	0.05	49.33

RBF SVR, radial basis function support vector regression (nonlinear support vector regression).

MLR, multiple linear regression.

Mean absolute difference is the average of the absolute differences between the predicted and measured values.

**Figure 4:**
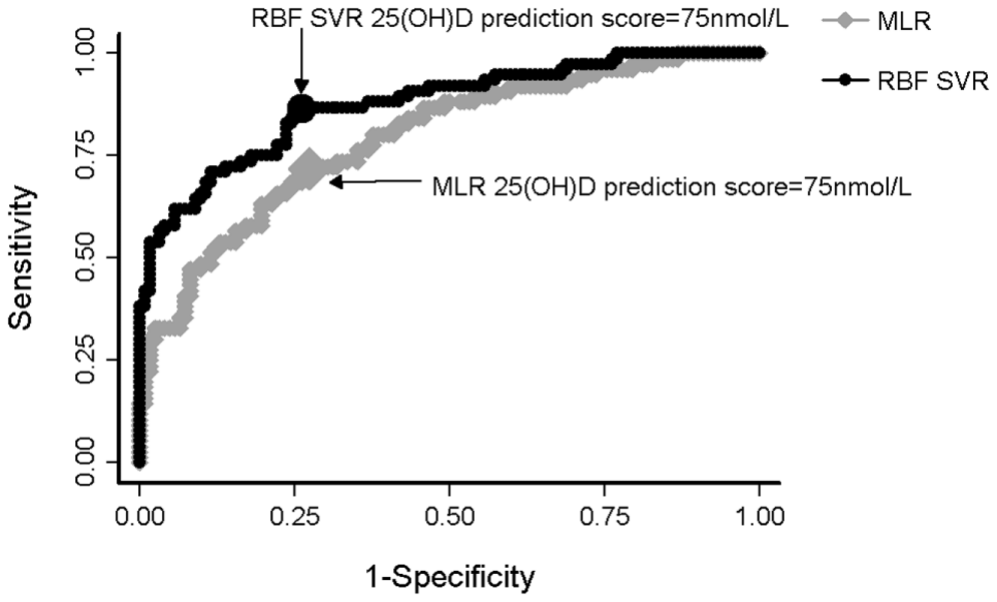
ROC curves of MLR and RBF SVR. ROC curves showing true-positive rates (sensitivity) plotted against the false-positive rate for different cut off points of the quantified components of MLR (gray diamonds) and RBF SVR (black circles). The points highlighted are 25(OH)D scores of 75 nmol/l for MLR and RBF SVR. The area under the curve is 0.79 and 0.87 for MLR and RBF SVR respectively.

We compared the sensitivity of the two modelling techniques for correctly classifying individuals as being vitamin D deficient vs. sufficient, using different cut-points. When vitamin D deficiency was defined as 25(OH)D level of <75 nmol/L (vs. ≥75 nmol/L), both models had reasonable sensitivity, but the RBF SVR model performed significantly better (P<0.01, Figure 4). The sensitivity for the RBF SVR model was 81.6% compared to the MLR model of 67.1%. The area under the curve (AUC) for the MLR ROC curve was 0.79 (95% confidence interval (CI) 0.73–0.86) compared with an area under the curve of 0.87 (95%CI, 0.82–0.92) for RBF SVR. Using a 25(OH)D level of 50 nmol/L as the cut off point, the AUC for the MLR ROC curve was 0.79 (95%CI, 0.68–0.89) compared with an AUC of 0.86 (95%CI, 0.79–0.94) for RBF SVR, P = 0.064. Notably, however, only 13% of the test sample were vitamin D deficient according to this cut off point (25(OH)D <50 nmol/L) with 25(OH)D levels measured using an LC-MS/MS assay. The superior performance of the RBF SVR model was less apparent with the limited number of ‘positive’ cases. As previously reported, 25(OH)D levels from a Diasorin Liaison assay were also available for these samples[34] with the results negatively biased compared to results from the LC-MS/MS assay, i.e. a greater proportion of the sample <50 nmol/L. We thus also tested the performance of the two modelling methods using the Liaison 25(OH)D results. Here the AUC for the curve generated from the MLR results was 0.69 (95%CI, 0.62–0.76), compared to that for the RBF SVR of 0.83 (95%CI, 0.77–0.89). That is, the RBF SVR model performed significantly better than the MLR model, P<0.0001.

**Figure 5:**
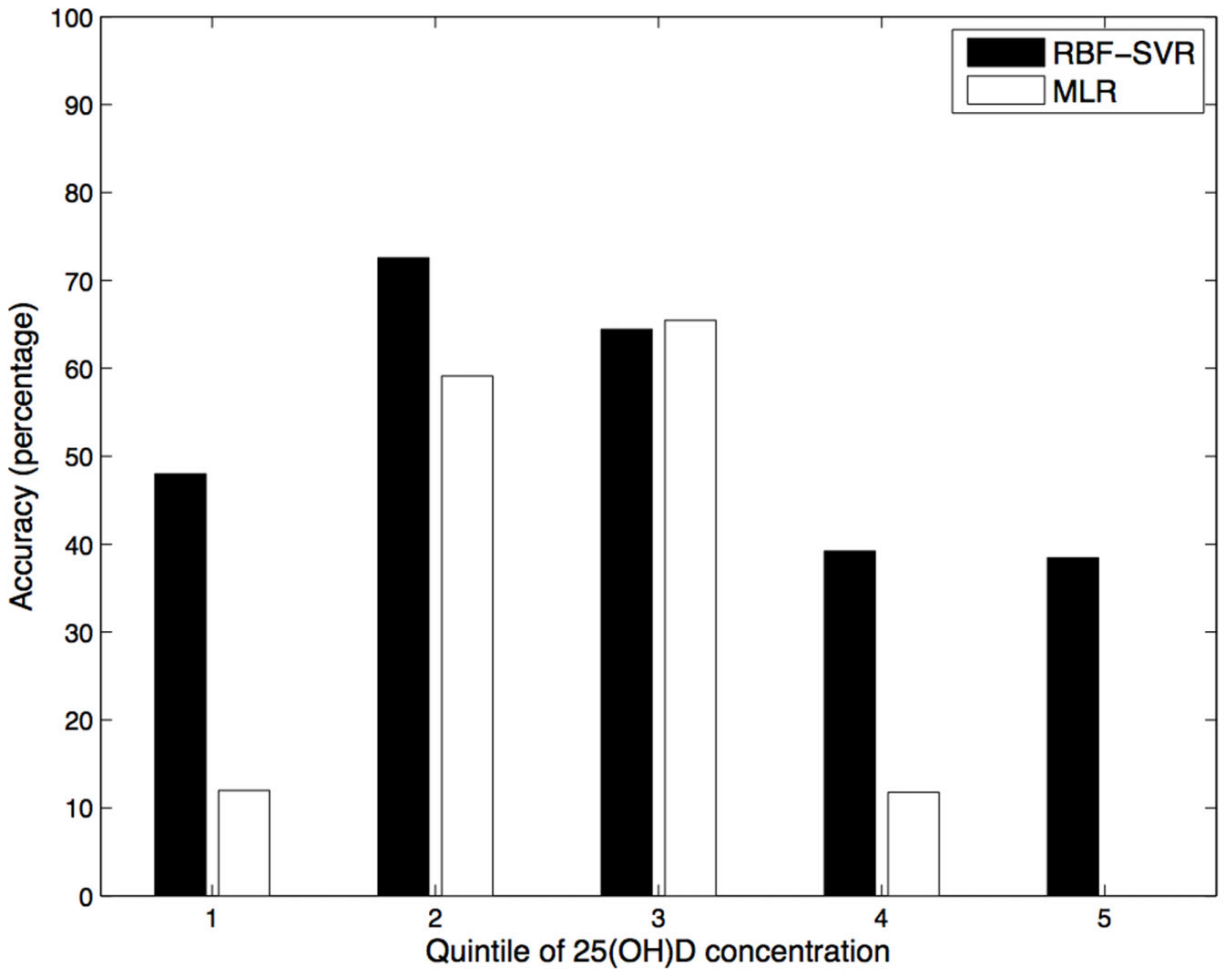
Accuracy of predicted 25(OH)D score in each quintile of 25(OH)D concentration.

**Figure 6:**
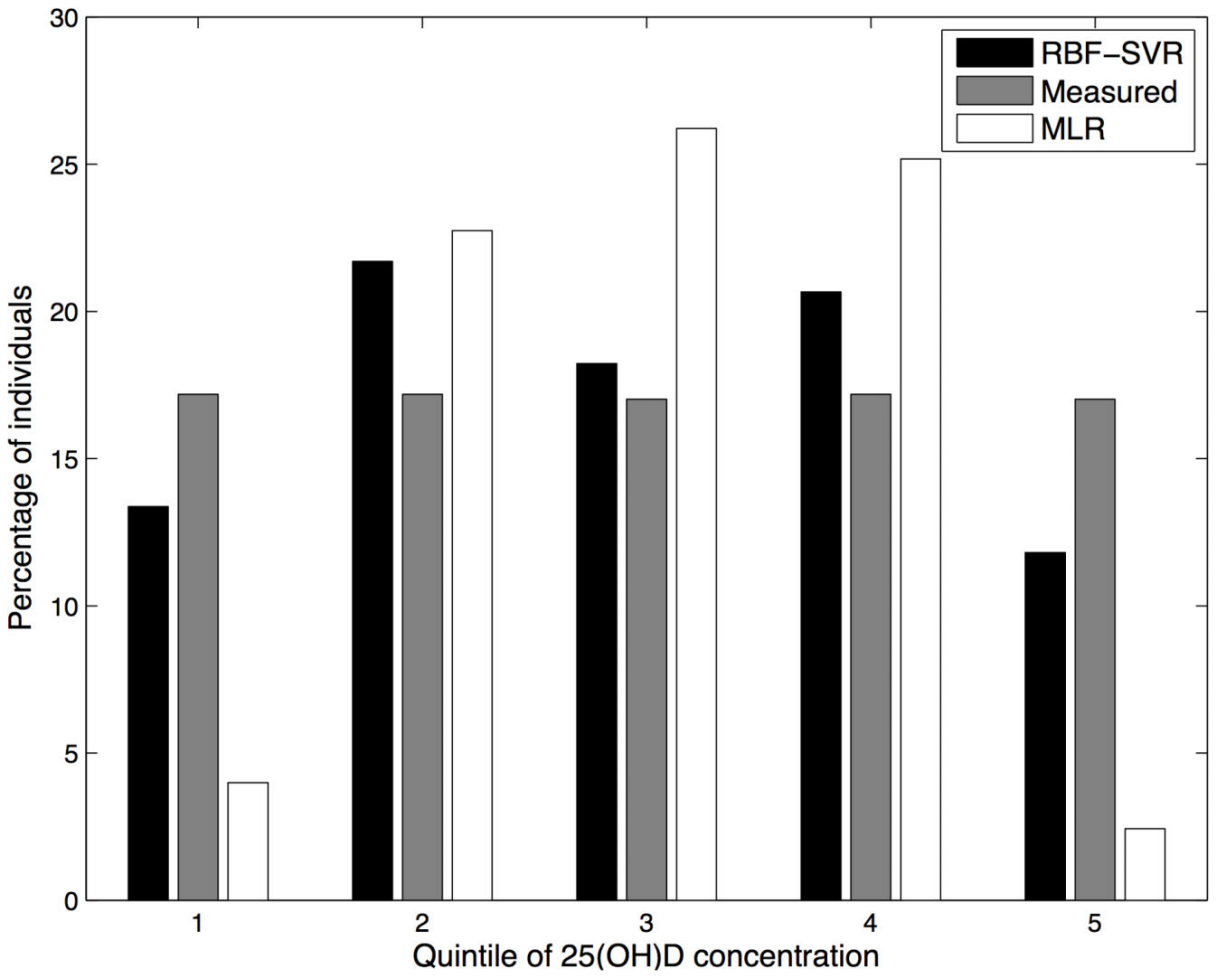
Percentage of individuals classified by quintiles of measured 25(OH)D concentration and predicted 25(OH)D score.

In epidemiological studies, exposures are often categorised into quintiles for analysis, so we classified predicted 25(OH)D scores and measured 25(OH)D concentration by quintile to determine how well the two prediction models performed in each quintile group. For the MLR model 50.2% of the predicted 25(OH)D scores, compared to 66.1% of predicted scores for the RBF SVR model, fell into the same quintile as the measured 25(OH)D values. Figure 5 shows the percentage of correct classification in each quintile. As is illustrated in Figure 5, both MLR and RBF SVR models performed well in predicting 25(OH)D concentration in the second and third quintile (Q2 and Q3). Although both prediction models were limited in their detection of extreme values the RBF SVR model had superior performance compared to the MLR model for correct prediction in quintiles 1, 4 and 5. The MLR model had very poor performance in predicting the highest serum 25(OH)D score; the prediction accuracy for Q5 was 0%. Figure 6 illustrates the percentage of individuals classified into each quintile according to actual and predicted 25(OH)D concentration. The quintile distribution of predicted 25(OH)D concentration derived from RBF SVR model is much more accurate than the MLR model, according to the quintile distribution of measured 25(OH)D concentration.

## Discussion

We compared the performance of MLR and RBF SVR models for the prediction of vitamin D status, using a set of pre-determined explanatory variables. Using the RBF SVR for prediction of serum 25(OH)D concentration resulted in lower mean absolute error in comparison with the MLR model. In the validation sample we observed better correlation between predicted scores and measured 25(OH)D concentration for the RBF SVR model compared to the MLR model. Furthermore, the RBF SVR method demonstrated higher sensitivity in classifying vitamin D status as deficient/sufficient and the AUC for the RBF SVR ROC curve was significantly larger than that for the MLR ROC curve.

This is the first study in which serum 25(OH)D concentration has been modelled using RBF SVR, with previous studies focussing on MLR models. For example, Bertrand *et al* [10] reported a MLR model using data from three US cohorts, with Spearman correlation coefficients between predicted and measured 25(OH)D of 0.23, 0.40, and 0.24, respectively. In the Women's Health Initiative, Millen *et al*.[12] reported a comparable correlation (0.45), using a MLR model. In the Framingham Offspring Study, Liu *et al*.[9] observed a correlation of 0.51 between predicted and measured levels. Using the results from these prediction models imposes several limitations on the accurate estimation of “exposure” in chronic disease epidemiology. Such models have substantial unexplained variability (R^2^ = 0.13–0.42) and the predicted scores are only moderately correlated with actual 25(OH)D levels. In previous studies, the predicted scores were based on data that were incomplete for known determinants of vitamin D status, such as sun sensitivity characteristics (e.g. skin colour, ability to tan), actual sun exposure and sun exposure behaviours (e.g. time spent outdoors and protective clothing). Proxies such as physical activity and ethnicity were used instead of actual sun exposure and skin colour, allowing considerable measurement error and misclassification on key determinants.

In our study, time spent outdoors and direct measurements of untanned skin colour were included as predictors in the MLR model. But even so, the MLR model using these environmental and phenotypic factors explained only a modest proportion of the total variability in serum 25(OH)D levels (R^2^ = 0.36) and the Pearson correlation coefficient (for predicted vs. measured values) was 0.51. The performance of our MLR model was consistent with the prediction models reported in the previous studies, suggesting intrinsic limitations of the MLR models.

Here we did not use the R^2^ value to evaluate the performance of the RBF SVR model, because this method is not based on a least mean squares approach. However, using the RBF SVR model, we observed a correlation of 0.74 between predicted scores and measured 25(OH)D concentration. Moreover, the RBF SVR model had higher sensitivity and performed better than MLR in correctly identifying individuals with vitamin D deficiency. Interestingly, the difference in sensitivity and AUC between the two models was less when the prevalence of vitamin D deficiency was low, i.e. with a cut-point of 50 nmol/L using the 25(OH)D results from the LC-MS/MS assay.

Millen *et al*.[12] concluded that predicted 25(OH)D scores do not adequately reflect serum 25(OH)D concentrations, and Peiris *et al*.[13] argued that vitamin D status cannot be reliably predicted and that common laboratory tests are required, especially for high-risk groups. Our study indicates that 25(OH)D scores developed using an RBF SVR model much better reflect actual serum 25(OH)D concentration. Although the RBF SVR model had some limitations in predicting extreme values, generally, the estimated vitamin D status was consistent with the measured 25(OH)D concentration. One limitation of our analyses was that only one validation dataset was available. Future studies testing the RBF SVR model in a range of other populations would further advance the understanding of its utility as a tool in epidemiological studies. After validation in population-based datasets, tools developed from SVM models could also be of value to primary care physicians and others to assess the risk of vitamin D deficiency to provide a more rational basis for vitamin D testing.

## Conclusion

Our results demonstrated a statistically significant superiority of an RBF SVR model in comparison with a MLR model for the prediction of serum 25(OH)D concentrations in the Ausimmune Study dataset. The accuracy of 25(OH)D scores from the RBF SVR model was greater. Thus the RBF SVR method has considerable promise for the prediction of vitamin D status for use in chronic disease epidemiology and potentially other situations.
